# Hospital and Institutional News

**Published:** 1913-06-21

**Authors:** 


### HOSPITAL AND INSTITUTIONAL NEWS.

### ST. GEORGE'S HOSPITAL AND THE KING'S FUND.

The special meeting of governors of St. George's Hospital, which was held last week to consider the question of confirming the resolution of the house committee, passed on April 30, resulted in a further postponement. Mr. G. N. Turner opposed the resolution, and Mr. A. William West, treasurer of the hospital, moved an amendment: "That we should adjourn the further consideration of this question until we have the report of the committee which has been appointed at the request of the president, H.R.H. Princess Christian of Schleswig-Holstein." This amendment was carried by twenty-three votes to six. The report of the committee referred to is expected to be ready by the end of June.

### GLASGOW'S NEW EPILEPTIC COLONY.

The recent opening of the Glasgow Parish Council's Epileptic Colony at Stoneyetts, Chryston, gives to Scotland its first Poor-Law institution of the kind. There are eighty-six patients, or colonists, in residence, and eventually there will be six villa blocks with accommodation respectively for 150 of both sexes. It is interesting to compare the main points of difference between the Glasgow colony and that one at Monyhull, which must have served in more than one sense as its model. Mr. James Cunningham, chairman of the Council, remarked that the Monyhull institution consists of two-storeyed villas, and the Scottish institution of one storey only. The architect for the buildings is Mr. Robert Tannock, master of works to the Parish Council. Dr. Leonard Baugh, who is in charge of the colony, has been senior assistant medical officer at Gartlock Mental Hospital. Every opportunity is, of course, given to make the patients, as far as possible, self-supporting, and it is probably true that some of these patients might be able to support themselves in the world did not such Acts of Parliament as Employers' Liability and Workmen's Compensation inevitably restrict the chances of those more likely to incur risks than healthy persons. Representatives of the Local Government Board, of the Board of Lunacy, and of the voluntary hospitals have united in commending this important step in Poor-Law progress in Scotland. Monyhull may well be proud of the example which it has set. Other Scottish cities, we hope, will follow Glasgow's example.

### AFTER 180 YEARS: A RUMOURED REUNION.

The topic of the week in London institutional circles is a rumour, which we give with all due reserve and for no more than it is worth, of proposals for the amalgamation of Westminster Hospital and St. George's. Those who are well up in the history of London will remember that St. George's Hospital was founded in 1733 by a secession of some of the governors and the whole of the staff of the old Westminster Infirmary, of which the modern Westminster Hospital represents the non-seceders. More recently, in fact only a few months ago, a scheme was promoted, and is in act of realisation, for the removal of the Westminster Hospital to a less central district with a large population of the hospital class. Some seven or eight years ago St. George's entertained a similar scheme, which fell through for financial reasons. It will certainly be a remarkable achievement if these two hospitals really do join forces again after one hundred and eighty years of separation. The rumours to which we have referred are circumstantial, but it would be premature in the extreme to assume that any decisions have been taken either on the general principle or on details. Indeed we should have hesitated to mention them at all, except for the fact that they seem to have spread so far and so wide in institutional circles that it would be foolish to ignore them. The rumours may or may not be true, and the project (if such there be) may or may not come to fruition; but at least the romance, if it may be so called, of the mere suggestion arrests immediate attention.

### TWO HOSPITALS AT LOGGERHEADS.

An inquest at Birkenhead on a child who died as the result of scalds accidentally received as a consequence of falling into boiling water has revealed the existence of a serious degree of friction between the Borough and the Children's Hospitals. After the accident, which took place on Saturday, June 7, about 8 p.m., the child was taken to the Borough Hospital and treated, but not detained there. On Monday she was removed to the Children's Hospital, where she died within two days. The matron of the Borough Hospital, Miss Margaret Macmillan, in giving evidence, said that she did not consider the case serious enough to detain. In answer to the Coroner's question she said there was friction between the Borough Hospital and the Children's Hospital. In replying to further questions she said: "I think it is between the medical officers of the institutions, because the matron of the Children's Hospital and myself are good friends. There is friction in that every case we send up to the Children's Hospital has some little thing said about it, and it is not as if we sent cases without first letting them know by telephone." In addressing the jury the Coroner said: "This friction will not be removed by mere recriminations as to who is to blame; both parties will have to let bygones be bygones, and to say that the friction shall not continue." We agree with the foreman of the jury in hoping that the inquiry will lead to a readjustment of relations which as at present described must do incalculable harm to both institutions. Friction between hospitals cannot possibly be tolerated, and the public will expect the authorities to guarantee its cessation as publicly as its existence has been exposed.

### NEW SUPERINTENDENT OF NORTH WALES COUNTIES MENTAL HOSPITAL.

Dr. Frank Jones, the senior assistant medical officer, was elected medical superintendent of the North Wales Counties Mental Hospital on Monday. Dr. Jones thus succeeds Dr. Stanley

Hughes, who had resigned to take up a similar post at the Salop Mental Hospital. The commencing salary attached to the North Wales post is £500 a year, with house, etc. Dr. Evans, of Prestatyn, and Dr. Benson Evans, of Denbigh, having declined to be proposed for appointment as first and second medical assistants at the North Wales Counties Mental Hospital, the committee of visitors have decided to advertise the positions. It will be remembered that the delay in appointing a new superintendent produced a letter in our columns from an "Asylum Medical Officer" criticising the committee on the ground that they were determined to get a Welshman.

### THE HOSPITAL SITE AT HASTINGS.

The offer on the part of the Hastings Corporation of £15,050 for the present site of the East Sussex Hospital was considered at a meeting of governors last week, presided over by Lord Hythe. Councillor W. C. T. Beasley, who is chairman of the hospital committee, said that the committee were unanimously in favour of accepting the offer of the Corporation. It it were accepted, he added, they would have sufficient to justify them in starting on a new building. He moved a resolution authorising the committee of management and the trustees, at any time within six months, to enter into a contract to effect the sale. One of the conditions will be that possession cannot be given until the new hospital is ready to receive patients. Mr. A. E. Young seconded the resolution, which was carried. It appears that with the funds in hand, and the sum received from the present site, a balance of £6,000 will probably remain to be found for the cost of a new institution, to which, we may add, the study of the advantages of open-air wards, as referred to elsewhere, may be commended.

### CHANGES AT THE PHARMACEUTICAL SOCIETY.

It is of interest to note that both the new president and the new secretary of the Pharmaceutical Society have had considerable experience of the pharmaceutical side of institutional work. Mr. Edmund White, B.Sc., F.I.C., the new president, was for many years pharmacist to St. Thomas's Hospital, where he was able to introduce many improved methods of manufacture by which economies were effected; while at the hospital he applied the principles of asepsis in the treatment of dressings, being the first British pharmacist to undertake work of this kind. His intimate knowledge of hospital pharmacy will be useful to him in his new position in dealing with matters which are not unlikely to arise during his period of office. Mr. W. F. Uglow Woolcock, the new secretary and registrar, is a pharmaceutical chemist and a barrister; he was for eight years in the pharmaceutical laboratory at University College Hospital. Mr. Woolcock is also a member of the Advisory Committee to the National Insurance Commissioners and secretary of the Pharmaceutical Standing Committee on Insurance. Mr. Richard Bremridge, who has retired from the position of secretary and registrar, had been in the service of the Pharmaceutical Society for forty-five years.

### THE PROPRIETARY MEDICINE EVIL: A SUGGESTED REMEDY.

A definite suggestion has been made to the Select Committee on Proprietary Medicines for dealing with fraudulent manufacturers. Mr. Guy Stephenson, Assistant Director of Public Prosecutions, who, when he appeared before the Committee earlier in the inquiry, was not prepared to make any proposal, but has since consulted with Sir Charles Matthews, now suggests that legislation might be introduced to set up some machinery similar to that which is used for the enforcement of the Sale of Food and Drugs Acts. This would mean the appointment of a staff of inspectors, by local authorities, whose duty it would be to look out for the commission of offences with reference to proprietary medicines; to make systematic search of the files of the newspapers, and to note any advertisements therein of an extravagant or suspicious character; to purchase the medicine advertised, and to cause it to be analysed. On the result of the analysis would depend subsequent action. The suggestion appears to be good so far as it goes, but it is doubtful whether the method proposed would touch more than the fringe of the traffic.

### MEMORIAL TO A TUNBRIDGE WELLS PRACTITIONER.

A year or more ago Dr. J. E. Ranking, a well-known practitioner of Tunbridge Wells, was killed in a motor-car accident, as recorded at the time in The Hospital. A public appeal for subscriptions to provide a suitable memorial met with so generous a response that £1,273 15s. 8d. was collected. Dr. Ranking was senior physician at the date of his death to the Tunbridge Wells General Hospital, in which he was especially interested, and to which he had rendered long and devoted service. It has accordingly and most appropriately been decided to commemorate his life by setting aside £1,000 out of the memorial fund for the permanent endowment of a bed in this hospital. Part of the balance will be spent upon a medallion to be placed in the hospital, and the remainder of it will then be handed over to the Samaritan Fund. It would be hard to devise any better way of commemorating Dr. Ranking's ever-ready help and assistance.

### THE SETTLEMENT AT SWANSEA.

A settlement has been come to between the medical men and the trade unions and friendly societies in the Swansea district, whereby the crisis to the General Hospital, Swansea, has been averted. It will be remembered that a resolution of the medical staff not to treat members of medical aid societies provoked a threat from the working-classes that their subscriptions would be withdrawn. The medical men have agreed to a rate of 10s. 6d. a year for wife and children under sixteen of an insured person instead of the 13s.

which they originally asked for, but this will be a net sum, whereas management expenses were to be deducted from the higher figure. They have agreed also to give all ordinary certificates free of charge, while Clause 5 of the terms of settlement that "the services to be rendered by the doctors shall conform to the regulations laid down in the Insurance Act" is interpreted as involving no extra charge for operations and anæsthetics. Free choice of doctor from among those on the panel willing to serve, and free choice of patients from those desiring his services are also among the terms of settlement. The settlement has been made possible by the understanding that the labour organisations bind themselves to oppose the formation of any medical aid society in the district, and to use their influence to break up those already formed, while the staff of the hospital agree to hold in abeyance "for a reasonable period," say, till the end of the present year, their recent resolution refusing to treat patients from any medical aid society. Great credit is due to the Mayor, Mr. David Williams, for the prominent and active part he has taken in bridging the difficulty and securing its peaceful solution.

### STAFF DIFFICULTIES AT A LONDON INFIRMARY.

Dr. A. Lionel Baly, medical superintendent at Lambeth Infirmary, again presses the Guardians in his annual report to bring the institution up to date. In the first place Dr. Baly points out that the mortality has shown a very considerable increase, so that the work in the wards is steadily rising. The number of patients on the danger list is over 100, which is unprecedented; and he points these statements by remarking that the average patient in an infirmary requires more attention than the patient in a general hospital. The staff, therefore, should be equal in number, and then, he adds, the difficulties might be decreased if the Board pressed forward the relaying of the floors and the removal of the antiquated beds with wooden frames. The Board, as a first step to remove the difficulties, has decided to refloor six wards, and to purchase 144 bedsteads. We congratulate Dr. Baly on the success which has crowned his pertinacity in pressing the need for these reforms.

### RATE AID AND THE VOLUNTARY SYSTEM.

The festival dinner of the Prince of Wales's Hospital, Tottenham, was held last week, and gained added importance from the fact that the example of Liverpool in obtaining Parliamentary powers to make contributions to its voluntary hospitals was made the chief point in the speeches. In proposing "Prosperity to the hospital," Sir Henry Burdett urged that it was the duty of the London County Council to follow Liverpool's example and contribute to the hospital. He emphasised the fact that his proposal did not mean that the hospitals should become rate-supported institutions. The State could only demand to inspect the wards and had no right to interfere with the management. England, he continued, would never allow the voluntary system to be replaced by one in which the patient became mere material for science. The present needs of that hospital were more income, more beds, and much more capital. Readers of our article in The Hospital of June 14, on "Municipal and County Council Grants for Voluntary Hospital Construction," with special reference to the policy which the Liverpool Corporation has received powers to carry out, will understand how "grants made by municipalities and County Councils to voluntary hospitals for building purposes on the lines followed first in Manchester and now at Liverpool should help and not hinder the perpetuation of the voluntary system." What are our readers' views?

### THE SOUTH LONDON HOSPITAL FOR WOMEN.

Princess Louise, Duchess of Argyll, will lay the foundation-stone of the South London Hospital for Women on Tuesday, July 1. The hospital, which is to be officered entirely by medical women, is to be built on a site facing Clapham Common. Of the £55,000 required to build and equip the hospital, we understand that £40,000 has already been subscribed, and the board of management hope to raise the remaining £15,000 before July 1, so that the hospital may be free of debt when the foundation-stone is laid.

### THE PASSING OF THE CLOSED WARD.

A change is sweeping over hospital construction. It began with the movement for country hospitals, and the taking away, wherever possible, of the ward pavilions from the towns. It is becoming the substitution of the open ward for the ward pavilion, not merely for children's hospitals or sanatoria, but for the treatment of diseases generally. We have already the Liverpool Country Hospital for Children at Heswall, Cheshire, which was one of the pioneer institutions in this respect in the North. The same principle is being recognised in Ireland, and only last week Sir Lambert Ormsby, senior medical officer to the National Children's Hospital, Dublin, urged the importance of a country hospital. He remarked that his long experience in the treatment of children's diseases had impressed on him the hopelessness of treating extensive tuberculosis disease of bones and joints in children in the closed wards of a city hospital. He urged the committee to collect funds to build a children's country hospital. The present building should be used, if possible, only as a receiving hospital, and the children after examination should be passed on in batches to the country hospital to be supplemented by open-air treatment. Now, although a few children's hospitals have been the first to make open-air treatment as much a matter of routine as in sanatoria for tuberculosis, it is manifest that it is not only diseases of the lungs and of the joints which benefit by open air. In their degree almost all diseases benefit by open air, as much as wounds by cleanliness, and the day is coming when the ward block as one knows it to-day will be modified generally in this respect. The adoption of open-air treatment for disease generally would have an important economic effect on construction bills, for the cost per bed would be much reduced wherever the closed ward is abandoned in favour of what may be described as the verandah principle.

### FURTHER GIFT TO MIDDLESEX HOSPITAL.

The new pathological block and institute of hygiene which was recently stated to be promised to the Middlesex Hospital, thanks to the gift of £10,000 from a private donor, is now to be extended both in capacity and in equipment. This extension is due to the same donor's promise to increase his gift to £15,000.

### THE CONSTITUTION OF THE SUNDAY FUND.

A special general meeting of the constituents of the Metropolitan Hospital Sunday Fund is being held on Friday, June 20, after we go to press, at the Mansion House. The business before the meeting is to consider the amended laws of the Fund's constitution (see The Hospital, May 24, 1913, p. 259), which have been drawn up as a result of the Council's resolution passed on January 29 last. The Lord Mayor, Sir David Burnett, the president of the Fund, is taking the Chair.

### INSTITUTIONAL ASPECT OF MEDICAL DEFENCE

The annual reports of the Medical Defence Union and the London and Counties Medical Protection Society contain the usual striking illustrative cases of the enormous value to medical men of membership of these defence societies. Both of them are continuing to increase both in membership and in financial strength; but it may be noted that numerical increase in both of them in 1912 was below the average of previous years. We cannot imagine that any young man beginning practice would neglect to join one or other of them, if only his attention were drawn to the matter sufficiently; possibly the percentage of the profession on the lists is now so high that only the newcomers afford any longer a field for harvesting. From an institutional aspect, medical defence has a value of its own. The exact legal responsibilities of an institution for acts committed by doctors who are members of its staff has never been authoritatively laid down, though isolated decisions upon particular points have been given. It is, however, very greatly to the advantage of an institution that any action against either the governors or any individual member or members of the professional staff should be defended by such experienced lawyers as those who advise these societies; and for our own part we think a hospital doctor neglects the interests of his institution as well as his own if he does not belong to one or other of these admirable societies.

### DR. DABBS' LAST DIARY.

An inquest was held on the body of Dr. G. H. R. Dabbs, who, as we stated last week, died suddenly, aged sixty-seven. The evidence showed that he was found by his housekeeper, and his partner, Dr. Alexander Simpson, said that he had suffered from angina pectoris, the intense pain of which he would alleviate by inhalations of chloroform. By the side of his bed were found an empty chloroform bottle and a piece of paper with notes headed "My case." This was in the form of a diary of his illness for the Saturday and Sunday preceding his death. He was found at midday on Tuesday, June 10. The notes are of personal rather than medical interest, and those headed Sunday, June 8, are an example: "Bad night. Oh the humiliations of disease! Shall keep quiet all day, and die like a stag if I may be alone. I have avoided morphia hitherto, but the pain is too much, and chloroform is like milk to me. I do so want to live! The world is so full of light, and life, and interest. Jack Seely will do if he will only remember his own explanations, but will he? If Winston had been in opposition now he would have been Prime Minister in six months." There is an obvious sign here of wandering, but it was very characteristic of his itch for writing to attempt notes of "the patient's point of view" in what proved to be his last illness. Had he recovered, probably the notes would have been used for the "Journal" which he used to publish. A verdict of "Death by misadventure" was returned.

### NEW COTTAGE HOSPITAL AT HENDON.

Princess Henry of Battenburg opened, last Saturday, the new Cottage Hospital at Hendon which has been erected as a King Edward Memorial. The site was given by Sir Audley Neeld, and the institution has been built at a cost of £2,500, and, what is also deserving of mention, has been opened free of debt. The honorary secretary of the hospital is Mr. J. Anderson. Princess Henry was received at the institution by Sir Audley and Lady Neeld.

### THE VALUE OF ELECTRIC CLOCKS.

A contract has been signed for the supply of electrically controlled clocks throughout the London Hospital. At present it contains 169 clocks, and it was felt that the time had come both from an economical and an efficiency point of view to place them under central control. It is interesting to note, though in another connection, that the cost of the tablet recently unveiled to the memory of John Harrison, the founder of the London Hospital, was defrayed by the members of the Committee of the London Hospital in testimony of their feeling that there should be a permanent record of so great a benefactor, and that the expenditure incurred could not reasonably come out of the funds of the hospital. The larger the institution the more centralising is undertaken, and electrically controlled clocks are becoming the rule in England as elsewhere.

### "UNIVERSAL SPECIALISTS."

A long string of weight-carrying medical names appeared last week under a letter advocating scientific study of mental defectives, and addressed to the editor of *The Times*. Among the signatories there was not to be found a single alienist. This fact reminds us that when the late Lord Acton introduced the late Dr. Emil Reich to an assembly of historians, they say that he observed—" Gentlemen, I bring you an universal specialist." One or two leaders of the medical profession do their best, and do indeed something, to come up to this description, but they themselves would hardly claim to answer to it. We have plenty of able men in lunacy; well, let them speak for themselves—if only to disabuse British minds of the idea that the man distinguished in some parts of the now vast subject of medicine necessarily knows all about it. It is a mischievous idea enough. We knew of a case where an ecclesiastic obtained a tropical colonial bishopric simply because a rather eminent surgeon had told him the climate would be good for his son's consumption. Within a few months the disease became active again, and the boy had to be sent home. There is no gross error like this involved in the present instance; in fact, the contention is correct enough; but general physicians, and even pathologists, are foolish nowadays not to submit to that decentralisation of medical knowledge which increases every year. There is very little medical publicism without Sir James Crichton Browne having a hand in it; and it is curious to find his name absent when he has claims to special knowledge of the subject. We repeat, let these institutional officers speak for themselves.

### WOMEN RESIDENTS IN ISOLATION HOSPITALS.

A scarcity of women doctors has led the Stoke-on-Trent Joint Hospital Board to raise the salary recently offered by them in an advertisement for a resident woman medical officer at Bucknall Isolation Hospital from £100 to £150. In an amusing speech, Dr. R. H. Read, the chairman of the board, recently stated that with a salary of £100 they could not expect to obtain experienced women doctors, and if they were to expect young girls with high spirits to consent to shut themselves off from the world in an isolation hospital they must tempt them with a higher salary.

### THE VALUE OF LADIES' ASSOCIATIONS.

The Gravesend Hospital has recently benefited by an elaborately organised fête, known as the "Olde Village Fayre," which has achieved a remarkable result. The profit reaped by the hospital is £1,155, which, Mr. C. E. Hatten has pointed out, is sufficient to pay off the debt of the last two years and to meet the adverse balance which is expected. This result has been achieved only by the hard work of the organisers, especially Mr. W. Pearson, the secretary, and Mrs. Peyton, the chairman of the Ladies' Committee, while to Lord and Lady Darnley's good offices is attributed the presence of Royalty at the "Fayre." The Ladies' Association appears to have been primarily responsible for the holding of the "Fayre," and we gladly mention this fact as only the latest example, to any hospital which still needs a reminder, of the immense value which such associations prove to the institutions which encourage them. It is not only in their specific duties that their worth is seen, but, as this instance perhaps serves to show, in the field of ideas which they supply. That a Ladies' Association may lift the weight of a three-years' debt from a hospital seems a great achievement, but that has been the result of such efforts at Gravesend.

### CONTROL OF PUBLIC HEALTH IN WALES.

A serious difficulty in connection with the Insurance Act has arisen in Wales. The Pembrokeshire County Council has adopted a report of its health committee in which certain difficulties in correlating the powers and functions of the Association for erecting a National Memorial to King Edward VII. and of the Welsh County Councils are brought to light. The Councils desire to be in their own districts the chief health authorities in Wales. The Memorial Association aims at the erection of sanatoria and at an anti-tuberculosis educational campaign. Both the Association and the Pembrokeshire County Council made an agreement in which the Association was to provide, control, and manage consumption sanatoria, subject to its receiving capital grants from the Treasury, and also the cost of their maintenance. The health committee says that the Memorial Association now submit an estimate for the current year and ask for the proceeds of a ½d. rate. But the *crux* arises over the twelve tuberculosis physicians which the committee agreed that the Association should appoint. The Association desires to be independent. The Local Government Board desires these physicians to be administratively subject to the county medical officers, a desire with which the Association's representatives recently refused at Shrewsbury to comply. The report of the health committee remarks: "We feel that once the County Councils allow their powers to be taken from their hands the powers will be irrecoverable, and that in our future Acts of Parliament the Association, which by its charter has power to deal with tuberculosis *and other* diseases, will be recognised as the chief health authority for Wales?" Would not this be an anomalous position for a voluntary body?

### A LONDON INFIRMARY'S SKIAGRAPHS.

Dr. W. L. Maccormac, medical superintendent of St. James' Infirmary, Wandsworth, which was only opened in June 1911, calls attention in his report to the results of the *x*-ray department, worked by Mr. Kinsman, the dispenser, which he describes as excellent; a total of 185 examinations were made on 143 patients. The institution is gradually accumulating an indexed series of most interesting skiagraphs, and has shown a praiseworthy example in indexing them from the start.

### A DISPENSER'S QUALIFICATION.

The main features of the proposed Bill to establish a qualification for dispensers has now been disclosed. The object of the measure, which is being drafted by the Pharmaceutical Society with the approval of the General Medical Council, is to set up a register of assistant dispensers. The Bill will provide for the inclusion in the register of persons who immediately prior to December 16, 1911, had acted for three years as dispensers to a medical man or a pharmacist in a public institution. Provision will also be made for the inclusion of certified apothecaries' assistants, and for the establishment of an elementary examination in prescription reading, posology, and pharmacy. All the authorities representing the parties interested will be consulted before the measure is introduced into Parliament, so that little opposition is anticipated.

### THE MEANING OF WEALTH.

In these days of large fortunes and, on occasion, of large gifts to charities it may seem superfluous at first sight to ask: What is wealth? This word is interpreted by the majority as meaning riches, that is money; but this is far too limited a definition. The wealthy in the popular sense are undoubtedly those persons believed to be fortunate who possess more than enough of this world's goods to meet their own personal requirements. But wealth in the sense we wish to use it means the possession of, or at least the opportunity to possess, all the higher moral and intellectual qualities which constitute a man or a woman at his or her best. Those persons of both sexes who are wealthy in the highest and best meaning of the word have heavy responsibilities, because the larger the intellect and the higher the standard of individual conduct, the greater is the influence for good which each of them can exercise in daily life throughout the whole period of their residence on this planet. The recent appeal on behalf of Guy's Hospital's Medical and Dental Schools, which Mr. Balfour upheld so well in the speech which we published, gave to the public an opportunity to appreciate the distinction between wealth and riches. The former is represented by the enthusiasm, skill, and self-sacrifice which has achieved the building of the new schools on the part of the staff and friends of the hospital. To the latter, to riches, is now given the opportunity of supporting with capital the work which wealth, as properly defined, has built up.

### EDDOWES v. ST. JOHN'S HOSPITAL.

On Monday afternoon an important action began in the Court of King's Bench which is likely to prove of great interest to institutional officers. Known as Eddowes and Others *v.* St. John's Hospital for Diseases of the Skin, the point at issue is alleged wrongful dismissal of a member of the honorary medical staff. The result of the action, which has been pending for about two years, is expected to decide what are the powers of a board of management to terminate the appointment of a member of the honorary medical staff. As we go to press the action is still in progress.

### IRISH DOCTORS AND THE INSURANCE ACT.

At the annual general meeting of the Irish Medical Association last week, with Dr. J. B. Story, the newly elected president, in the chair, a resolution that "the representatives of the Irish Medical Association on the Conjoint Committee be called upon to resign their positions on that Committee" was carried almost unanimously. It was also resolved that all members of the Association who have gone on the panel in their areas and outside their areas to certify for insured patients attended by non-panel doctors be called upon to resign their membership of the Association. The net result of these resolutions appears to be a complete deadlock over the question of medical certification, with the effect of making the "benefits" of the Act non-existent. The demand for the exemption of Ireland from the scope of the Act is therefore already made, on the ground that it is the only "amendment" of the Act which would satisfy the public or the profession.

### MR. GUNTRIP KING'S SUCCESSOR.

The new secretary of the Samaritan Free Hospital, who fills the post which recently became vacant through the death of Mr. W. Guntrip King, is Mr. Paterson. We believe that this new post is the second promotion which Mr. Paterson has had since January. He comes to the Samaritan Free Hospital from the Royal Dental Hospital, where he has been assistant secretary. Mr. Paterson was for some years assistant secretary at the offices of the Royal Society of Medicine. Among the candidates who appeared before the selection committee of the Samaritan Free Hospital were the assistant secretaries of Queen Charlotte's Hospital, Great Northern Central Hospital, Charing Cross Hospital, and Queen's Hospital for Children, as well as Mr. H. Coope, who has been acting secretary since Mr. Guntrip King's death.

### THIS WEEK'S DRUG MARKET.

Several changes in value have taken place during the week, in some instances the change being in a downward direction. With the exception of the demand for such articles as tartaric and citric acids and essence of lemon, business in the Drug market continues rather dull. Tartaric acid tends upwards in price owing to shortage of supply, and the extreme value of essence of lemon is well maintained. Cream of tartar is also in good demand. It is thought probable that the price of cod-liver oil is now at its lowest limit, and there is, in fact, a tendency towards an upward movement; there is naturally not much business being done in the oil at the moment, but it seems not unlikely that when the demand sets in again prices may advance. Opium is rather dearer in the primary markets, but there is no change here, and it is not altogether improbable that prices will be lower soon. The price of camphor may possibly be reduced slightly as a result of a small reduction in the value of crude. Quinine is unchanged in price. Gentian root and senega root are both tending somewhat dearer. Ammonium carbonate is slightly cheaper. At the recent public sale of drugs in Mincing Lane lower prices were accepted for ipecacuanha, sarsaparilla, aloes, and cardamoms.

